# Coroner’s Investigation.—Suspected Poisoning

**Published:** 1860-12

**Authors:** 


### CORONER'S INVESTIGATION,—SUSPECTED POISONING.

### CASE OF MARY E. ROSELL, OF BINGHAMTON, N. Y.

We copy the following from a reliable report in the Binghamton *Democrat.* The facts are worthy of record in several aspects. First, they show that the bi-chloride of mercury can be detected some time after death in the structures of the stomach and intestines, when no trace of it can be found in the contents of those organs. Second, they render it probable that the bi-chloride of mercury may be given in small and frequently repeated doses in a diluted state, and impregnate the tissues of the abdominal viscera sufficiently to preserve them from decomposition for a long time after death, and yet without leaving any traces of corrosive action on the mucous membrane. Third, they show to what absurdities the Homœopathic principle of "similia similiabus curanter" will lead its votaries.

The two last physicians sworn, Brown and Hand, are well known Homœopathists. The one had regarded the deceased as having had "nursing sore mouth," and the other as having had "inflammation of the mucous membrane of all the cavaties." Hence, both agreed that the disease consisted in an extremely tender and inflamed condition of the mouth, stomach, and intestines ; and yet on the principle that, *like* cures *like*, both prescribed the most irritating preparations of mercury, that are known to the profession, namely, the Iodide and the Bi-chloride or *Corrosive Sublimate.* The latter being given during the last two days of the patient's life. True Dr. Hand testifies that he "intended to administer about the four hundredth part of a grain at a dose." The post-mortem, however, shows that enough was given to impregnate all the tissues of the stomach and intestines. We have known a case in this city in which a prominent Homœopathic practitioner gave to a child a solution of Corosive Sublimate every day for more than a week, for the cure of dysentery complicated with ague; and another case in which another Homœopathist gave a solution of *arsenic*, to a delicate nursing infant with cholera infantum. Yet these are the class of doctors, of whom the community say "they certainly wont do any harm if they dont do any good":

Coroner Brigham, in accordance with the urgent request of many relatives of Mrs. Rosell, caused her remains to be exhumed on the 12th day of July last, and empanneled the following named jurors—Dr. E. G. Crafts, Dr. P. Brooks, W. A. Cole, A. G. Avery, Charles N. Fancher, Levi S. Hodge, and H. B. Ogden. The jury having inspected the body, reported to the Coroner their inability to determine the cause of death, and requested a *post-mortem* examination to be instituted. In conformity with this recommendation the Coroner employed and directed Dr. J. G. Orton to make the necessary *post-mortem* examination, and the jury were released subject to the call of the Coroner. On the 12th ult., the jury were summoned at the office of Dr. E. G. Crafts to take cognizance of the following testimony.

Dr. J. G. Orton, being duly sworn, deposes and says, that he resides in Binghamton, N. Y.; that he is a regular practitioner of medicine and practical chemist in that place; the deponent further says, that he was requested by E. W. Brigham, Coroner for the County of Broome, to make a *post-mortem* examination of the body of Mrs. Mary E. Rosell, on the 12th day of July last, between the hours of 9 and 12 o'clock A. M.; in accordance with his request, the Coroner employed Drs. E. G. Crafts and P. Brooks, as assistants in the *post-mortem* examination. The deponent further says: in consequence of the extensive general decomposition of the muscular tissue of the limbs (42 days having elapsed since death took place) it was impossible to determin whether *rigor mortis* had existed or not; the skull having been removed at its upper part, exhibited the brain in a complete state of decomposition; no traces of disease nor its absence could be discovered; the thorax being opened presented the lungs and heart both in a fair state of preservation, the former of a dark slate color, mottled in patches in its external surface; the substance of the lungs upon after examination, exhibited all of the characteristic signs of healthy condition, being of a light, porous, spongy texture, floated in water, crepitated when handled, and were elastic; there were no evidence of organic disease whatever; the heart was found in its normal position, and in size measured 4½ inches in length, 3½ in the broadest part of its transverse diameter, and 2½ inches in its antero-posterior; it weighed nearly nine ounces. Upon laying open the four cavities of the heart, the walls were found slightly thinner than natural, but nothing remarkably so; its structures was well preserved even to the *chordæ tendenæ and columnæ carnæ;* the right or anterior ventricle was normal in capacity; the tricuspid value, guarding the auriculo ventricular orifice, and the semi-lunar valves, that of the opening of the pulmonary artery, were entirely free from disease or evidences of former difficulty; the right auricle was in every respect normal in all of its parts; the left auricle was likewise in appearance; the left ventricle with its aureculo ventricular and aortic openings, guarded by the mitral and semi-lunear valves, exhibited no traces whatever of recent or remote disease; there were no pleural adhesions; the abdomen being laid open, exhibited the various viscera in a remarkable state of preservation; the peritoneum was characterized by its healthy appearance with no signs of recent inflammation; it was firmly connected with various portions of the abdomen by adhesions, evidently of long standing and not referable to any recent inflammation; these abnormal attatchments were principally confined to the right and left lumbar regions, but extended up to the umbilical; its reflections or folds forming the greater and lesser omentum were not characterized by any peculiarities worthy of note, except that of normal proportions; the same is also true of the mesentery. The stomach and intestines with their contents, the lungs, heart, liver, womb, kidneys, and bladder, after examination, with no apparent signs of disease, were placed in two new and carefully cleaned jars, covered and sealed with four seals, in the presence of the jury, and conveyed by the Coroner to my private laboratory, at my residence, corner of Henry and Canal streets, and placed under lock and seal, with a written direction from the Coroner to institute a thorough chemical analysis of the various parts, and to determine as medico legal evidence the presence or absence of poison.

The deponent further says: in accordance with the direction of the Coroner, I proceeded to examine the various parts of the body entrusted to my care; I found the liver of a redish brown color, mottled with spots of blue; it measured ten inches in breadth, six in length, two and a half in thickness, and weighed 2¾ lbs. Upon carefully removing a portion of the peritoneal or outer covering of the organ, an innumerable number of minute granules were found adhering to the inner or proper tunic; these granules were white and of considerable hardness, so much so as to give a gritty feel when the scalpel was scraped over them; they were found in almost every part of the parenchematous structure of the liver, wherever this membrane sends its processes; the hepatic duct was completely lined with them; examined under the microscope, they exhibited their true chrystaline character, namely, rhomboidal tables or plates, some of the angles were truncated; these granules were insoluble in cold water, but soluble in hot alcohol and ether, assumed a blood red color from the action of sulphuric acid; chemically examined, they proved to consist for the most part of cholesterine, fat globules and hippuric acid; the latter was soluble in cold alcohol, and deposited on evaporation beautiful crystals, some possessing a dentritic and plumose outline, while others were arranged like zeolites; minute needles mixed with four sided prisms accuminated at their ends were formed from a hot water solution; the kidneys, womb and bladder, were found in a healthy condition; the stomach was completely divided through its transverse diameter, and its contents consisting of a thick pasty substance of a bluish gray color, carefully removed for examination; the four coats of the stomach, namely, the serous or outer coat, the muscular cellular and mucous or inner coat, were perfectly preserved even to the minutest vessels and nerves; with the exception of a small portion of the mucous membrane at the lesser curvature of the organ, it was here of a darker hue, and separated upon scraping with the knife. The contents of the bowels were exceedingly slight, and found for the most part in the larger intestines; the coats of the intestines were also entirely preserved, not the slightest indications of decomposition were to be found upon close inspection.

(Dr. Orton here read to the Jury a complete history of the elaborate chemical analysis which he had made of the various portions of the body entrusted in his care, extending through a period of nearly three months, and the final results of the investigation were reported in the following concluding deposition.)

I found no traces whatever of the presence of mineral poison either in the fluid or solid contents of the stomach and bowels; I detected in every portion of the tissues of the stomach and intestines, the positive presence of the *bi chloride of mercury or corrosive sublimate*, and in quantity sufficient to account in my opinion for the remarkable state of preservation in which those parts of the body were found at the date of the post-mortem examination.

In concluding my report to this jury, I would call their attention to the following points for their special consideration.

1st. That this investigation has failed to establish as a cause of death, the presence of any organic disease of the important organs of the body.

2d. That the abnormal condition of the liver was of a peculiar nature, and undoubtedly indicative of derangement of the portal circulation, and the presence of these innumerable granules of cholesterine and hippuric acid beneath the peritoneal covering of the organ, and being the hepatic ducts, significant of the commencement at least, of that unfavorable diathesis, namely, a tendency to the formation of gall-stones.

3d. That murcury in the form of corrosive sublimate has been fonnd in every portion of the tissues of the stomach and intestines. This important fact will require at your hands the closest investigation. The absence of any signs of corrosion or ulceration in the stomach or intestines would indicate that it had been administered either in small and repeated doses or largely diluted. You will ascertain by the examination of the physicians who attended upon the diseased, whether corrosive sublimate or mercury in any form was administered as a medicine during her last illness, and also by the same witnesses and others, whether there were any symptoms manifested during her sickness or at the time of death which simulated poisoning by corrosive sublimate.

Dr. P. B. Brooks, being sworn, testified that he attended professionally upon Mrs. Rosell for a short time during the month of March last. Diagnosis, nursing sore mouth; administered quinine, decoction of peruvian bark, decoct. goldthread, sulphate of cinchonæ, chlorate of potash and borate of soda; no mercurials were given.

Mrs. L. Johnson, sworn, testified that she gave medicine sometimes, and had prescribed for the diceased several times since the 1st of January last, until about a week before she died; considered her condition due to ill treatment during her late confinement; gave her a decoction of various roots, and a wash for her mouth.

Dr. Lodowick Hanes, being sworn, testified that he had seen Mrs. Rosell only once professionally on the third of May last; the symptoms then exhibited, manifested those of *aphthæ*; administered opium with magnesia, and ordered mucilaginous drinks; during that visit a brother of the deceased having suggested the possibility of some poison having been administered to her; the deponent remarked that some of the symptoms did simulate the action of mineral poison.

Dr. T. L. Brown, deposed, that he had attended upon Mrs. Rosell sometime previous to her death. Diagnosis, nursing sore mouth; ordered sulphate of lime 3d trituration; continued this for three or four days, and then changed it for the iodide of mercury, 3d trituration, which was also continued three or four days; does not think death could have ensued if the whole amount administered had been taken at a single dose.

Dr. S. D. Hand, being sworn, testified that he was called to attend upon the deceased two days previous to her death. Diagnosis, inflammation of the mucous surfaces of all the cavities. Symptoms, pulse irritable, pain in the ears, intense thirst, soreness of the abdomen, a burning sensation in the stomach and bowels, at intervals a strong action of the heart, diarrhœa, occasionally alternating with vomiting, intervals of extreme prostration, and great palor of countenance; thought the disease might have been simple *aphthæ*, aggravated by injudicious or neglect of treatment; prescribed a solution of corrosive sublimate once in three hours in connection with some pellets of arsenicum; could not testify as to the strength of the solution of corrosive sublimate employed, but intended to administer about the four-hundredth part of a grain each dose.

VERDICT.

*State of New York,* }
BROOME COUNTY. } ss.

An inquisition indented and taken for the people of the State of New York, at the office of Dr. E. G. Crafts, in the town of Binghamton, in said County of Broome, commencing on the 12th day of July last, and which has adjourned to this date, Oct. 15th, 1860, before me, Elmer W. Brigham, one of the coroners in and for said county, upon the view of the body of Mary E. Rosell, upon the oaths of Edwin D. Crafts, Peletiah Brooks, Charles N. Fancher, A. G. Avery, Levi S. Hodge, Walker A. Cole, and H. B. Ogden, good and lawful men of the said county, who being duly sworn to inquire on the part of the people of the State of New York, into all the circumstances attending the cause of the death of the said

Mary Rosell, and in what manner, and when and where the said person came to her death, do say upon their oaths aforesaid, that the said Mary Rosell came to her death on the 31st day of May last, from a cause to them unknown.

In witness thereof, as well the said Coroner as the jury aforesaid, have to this inquisition set their hands and seals this 15th day of October, 1860.

| | |
|---|---|
| E. W. Brigham, Coroner, | C. N. Fancher, |
| E. G. Crafts, Foreman, | Walker A. Cole, |
| P. Brooks, | Albert G. Avery, |
| Henry B. Ogden, | Levi S. Hodge. |

